# CuNi Alloy NPs Anchored on Electrospun PVDF-HFP NFs Catalyst for H_2_ Production from Sodium Borohydride

**DOI:** 10.3390/polym15030474

**Published:** 2023-01-17

**Authors:** Ahmed Abutaleb

**Affiliations:** Department of Chemical Engineering, College of Engineering, Jazan University, Jazan 11451, Saudi Arabia; azabutaleb@jazanu.edu.sa

**Keywords:** chemical reaction engineering, nanofibers, electrospinning, sodium borohydride, hydrogen generation

## Abstract

Non-noble Cu_x_Ni_1−x_ (x = 0, 0.1, 0,2, 0.3, 0.4, 0.5, 0.6, 0.7, 0.8, 0.9, 1) alloy nanoparticles supported on poly(vinylidene fluoride-co-hexafluoropropene) (PVDF-HFP) nanofibers (NFs) are successfully fabricated. The fabrication process is executed through an electrospinning technique and in situ reduction in Cu^2+^ and Ni^2+^ salts. The as-synthesized catalysts are characterized using standard physiochemical techniques. They demonstrate the formation of bimetallic NiCu alloy supported on PVDF-HFP. The introduced bimetals show better catalytic activity for sodium borohydride (SBH) hydrolysis to produce H_2_, as compared to monometallic counterparts. The Cu_0.7_ Ni_0.3_/PVDF-HFP catalyst possesses the best catalytic performance in SBH hydrolysis as compared to the others bimetallic formulations. The kinetics studies indicate that the reaction is zero order and first order with respect to SBH concentration and catalyst amount, respectively. Furthermore, low activation energy (Ea = 27.81 kJ/mol) for the hydrolysis process of SBH solution is obtained. The excellent catalytic activity is regarded as the synergistic effects between Ni and Cu resulting from geometric effects over electronic effects and uniform distribution of bimetallic NPs. Furthermore, the catalyst displays a satisfying stability for five cycles for SBH hydrolysis. The activity has retained 93% from the initial activity. The introduced catalyst has broad prospects for commercial applications because of easy fabrication and lability.

## 1. Introduction

The fast growth in the world population is leading to an increase in global energy consumption that is primarily formed from fossil fuels (e.g., petroleum oil, coal, and natural gas). Combustion of fossil fuels produces toxic gases (e.g., carbon dioxide, nitrous oxide, etc.) that deteriorate the ecological environment [1,2,3]. Thus, the search for alternative and sustainable energy has become urgent worldwide. Hydrogen (H_2_) is considered one of the most hopeful energy carrier sources because of its high energy content and eco-friendly characteristics such as zero-pollutant emissions [4]. However, the practical application of H_2_ in large quantities (e.g., fuel cell vehicles) suffers from unsafe and inefficient H_2_ storage methods (e.g., compressed gasses or cryogenic liquids) at mild conditions, which is attributed to the low H_2_ density > 5 wt% [5,6,7,8,9]. In recent years, metal hydrides (e.g., sodium borohydride (NaBH_4_, SBH), lithium hydride (LiH), sodium aluminum hydride (LiAlH_4_), lithium aluminum hydride (LiAlH_4_), and lithium borohydride (LiBH_4_)) have been recognized as promising hydrogen reservoirs because of their extraordinary H_2_ storage density [10,11,12]. Amongst them, SBH is considered one of the ideal candidates for an H_2_ reservoir owing to its low density and low molecular weight, low cost, non-toxicity, and high hydrogen content (~10.8 wt.%). H_2_ can be generated through hydrolysis of SBH in the presence of appropriate catalysts like noble (e.g., Pt, Ru, and Pd) and non-noble metallic (e.g., Ni, Co, and Cu) nanoparticles (NPs) and their alloys [13,14,15]. Recently, bimetallic Cu-M (M=Fe, Co, Ni) with different geometries and compositions has become very attractive in the fast production of H_2_ from hydrogen storage materials [16,17,18,19]. However, NPs suffer from high agglomeration during H_2_ release as a vigorous hydrolysis reaction occurs and converts the NPs into regular particles [8,20,21,22]. To avoid the agglomeration issue, NPs were supported on different matrices such as carbon, silica, TiO_2_, zeolite, Al_2_O_3_, and others [23,24,25,26]. Ayman Yousef et al. [27] prepared NiCu nanorods@carbon NFs and found the superior catalytic activity for AB hydrolysis. Jun Zhang et al. [28] demonstrated porous hierarchical Cu@Ni cubic cage as a very active catalyst for AB hydrolysis. However, the catalysts in the powder form suffer from some issues such as (1) the separation of the suspension-powder NPs from the solution is difficult, (2) the NPs tend to coalesce and agglomerate, and (3) the suspension-powder NPs are difficult to apply on continuous flow. R. Fernandes et al. [29] supported NPs over thin films to avoid these issues. However, the fixation of NPs on the thin film decreased the NPs’ exposed surface [7]. Polymer NFs have been introduced as catalytic supports for numerous metallic and non-metallic NPs [30,31,32,33,34,35] and used in different chemical reactions [36,37,38]. Catalysts made with polymer NF supports are very easy to recycle and reuse efficiently. Additionally, NFs are well-known to have huge surface areas; most NFs possess double the surface area of continuous thin films [39]. Kim and his coworkers have prepared hybrid NFs composed from polyvinylidene fluoride (PVDF) and CoCl_2_ for H_2_ production from SBH [40,41,42,43,44,45]. In another work, they have prepared a PVDF/CoCl_2_/Y-Zeolite effective catalyst to generate H_2_ from SBH hydrolysis. The fabricated catalysts showed outstanding performance with fair chemical stability and reusability. The presence of regular and interconnected pores provides easy access for reactants and the release of products, evolved H_2_. It is well-known that fabricated NFs via a simple electrospinning technique can produce a highly uniform and interconnected pores structure within a large surface-to-volume ratio [20,46,47,48,49,50,51]. In recent years, electrospun nanofibrous PVDF-HFP mats have been fabricated and applied in different important applications such as a water treatment, polymer electrolyte, and catalytic support for different reactions [52,53,54,55,56]. PVDF-HFP is one of the most appropriate host polymers for making hybrid composites as compared to various host polymers. PVDF-HFP has excellent electrochemical and chemical stability with great affinity to absorb electrolyte solution. The attachment of metal NPs on the surface of PVDF-HFP NFs has dual effects, namely that it [57] (1) decreases the polymer crystallinity and (2) enhances the electrolyte solution uptake, which lead to an improvement in the SBH and catalyst surface contact. Accordingly, PVDF-HFP is an excellent catalytic support candidate to support metallic and non-metallic NPs.

This study aims to prepare Ni_x_Cu_1−x_ NPs-PVDF-HFP NFs as a bimetallic catalyst for H_2_ production from SBH hydrolysis. The fabricated NFs were prepared using an electrospinning technique followed by a chemical reduction process. Characterizations of the fabricated electrospun catalysts proved the fabrication of Ni_x_Cu_1−x_ bimetallic nanoalloy supported on PVDF-HFP NFs. The introduced NFs demonstrated excellent catalytic activity toward H_2_ generation from SBH. The fabricated Ni_0.7_Cu_0.3_/PVDF-HFP NFs exhibited the maximum catalytic activity. Furthermore, this composition showed good stability six times.

## 2. Materials and Methods

### 2.1. Materials

Poly(vinylidene fluoride-co-hexafluoropropene) ((PVDF-HFP), 98% assay, (MW = 65,000 g/mol)), nickel (II) acetate tetrahydrate ((NiAc), 98% assay), copper (II) acetate mono-hydrate ((CuAc), 98% assay), and sodium borohydride (SBH, NaBH4, 98% assay) were purchased from Aldrich Co., St. Louis, MO, USA. N, N-dimethylformamide (DMF, reagent grade, 99% assay), and acetone were brought from Fluka, Al-Khobar 31952 Saudi Arabia. All chemicals were utilized without any further modifications.

### 2.2. Experimental Work

All different proposed catalysts were fabricated through three consequence major steps: (i) preparation of electrospinning solutions, (ii) fabrication of polymeric NFs using electrospinning technique, and finally (iii) reduction in the catalytic acetate precursor into their metallic forms.

To prepare the electrospinning solutions, first, polymer solutions, 15 wt.% PVDF-HFP, were prepared by dissolving 1.5 g of PVDF-HFP powder in a solvent mixture of DMF and acetone (4:1). The polymeric solutions were mixed in a magnetic stirrer for at least 6 h to make very homogenous sol–gel solutions. Second, different composition from the catalytic precursors, NiAc and CuAc, were added to 20 g of the prepared homogenous PVDF-HFP solutions, separately. The amount of NiAc and CuAc were adjusted to fabricate NFs with Ni_1−x_Cu_x_ (x = 0–0.9). To ensure perfect dispersion of the catalytic precursors, the electrospinning sol–gel solutions were stirred, after adding the catalytic precursors, for 5 h at 50 °C. The NiAc-CuAc/PVDF-HFP solutions were finally cooled at room temperature to become ready to be electrospun.

The prepared electrospinning solutions were then electrospun using a lab-scale electrospinner to fabricate polymeric NFs with different catalytic precursors compositions. The electrospinning set-up consists of three major components: solution reservoir to hold the electrospinning solution, power supply to generate an electrical field, and a collector to collect the fabricated NFs. The fabricated polymeric solutions were placed in a reservoir, a 5 mL plastic syringe. The electrical field was generated by inserting a copper wire inside the plastic syringe from one end (positive electrode) while the other side, the collector, was grounded. The rotating drum collector was covered with aluminum foil to collect the fabricated NFs. The electrospinning operating conditions were fixed at room temperature and pressure, 20 kV and 18 cm distance between the syringe and the collector. Generally, most of the solvents evaporate while the NFs travel from the syringe to the collector; however, some residual solvent stays in the fabricated NFs. Hence, the fabricated NF mats were dried at 40 °C under vacuum overnight to ensure full evaporation of the solvent mixture.

### 2.3. Chemical Reduction in CuAc and NiAc Supported on PVDF-HFP NFs

To reduce catalytic precursors and convert NiAc and CuAc into their pure metallic forms, SBH was used as a reducing agent. Typically, to start the reduction process, first, 100 mL of methanol was placed in a 500 mL beaker. Then, the fabricated electrospun nanofibrous mats were immersed in the methanol solution. After that, SBH was added in the beaker and stirred at 1000 rpm. The molar ratios between metals precursor and SBH were adjusted at 1:5 to obtain a complete reduction reaction. To prevent vigorous reduction reactions, the reduction reaction was operated at low temperature. It was noticed that as soon as the fibrous mat was immersed in the solution, a gas bubble (H_2_) was produced with a green-to-black color change, indicating that NiAc and CuAc converted into Ni and Cu metals, respectively. The fibrous mat was kept in the SBH solution until no H_2_ bubbles were seen. The produced mat was washed by ethanol and DI water to eliminate impurities. Last, the reduced nanofibrous mat was dried overnight at 40 °C under vacuum.

Equation (1) shows that the methanolysis reaction produces a by-product, sodium tetra methoxy borate (NaB(OCH_3_)_4_) [8]. This by-product reacts with water during the washing process, as shown in Equation (2), and produces methanol and sodium borate, NaBO_2_, both of which are washed away with the excess water.

**NaBH_4_ + 4CH_3_OH → NaB(OCH_3_)_4_ + 4H_2_** (1)

**NaB(OCH_3_)_4_ + 2H_2_O → NaBO_2_ + 4CH_3_OH** (2)

It is worth mentioning that researchers usually use aqueous SBH to reduce metal salts [39]. However, SBH here was dissolved in methanol and used to execute the reduction step. In fact, aqueous SBH was initially applied as a reducing agent, and it was seen that the reduction reaction was much slower than the SBH/methanol reducing agent.

Using methanol instead of water provides important advantages. For example, the by-product, NaB(OCH_3_)_4_, does not rapidly polymerize into various types of polyborates. Preventing the precipitation of NaB(OH)_4_ is necessary to stop the poisoning of the catalyst [58,59]. Hongming et al. [60] used hydrothermal and reduction procedures to prepare ultrafine Co NPs@carbon nanospheres as an effective catalyst for the generation of H_2_ from SBH hydrolysis. Due to the insolubility of NaBO_2_ in ethanol, partial NaBO_2_ was precipitated out together with the Co NPs in the water media. This could separate the Co nanoparticles and further prevent them from agglomerating into larger clusters. They found that the reduction in Co ions in the ethanol solution media was more effective than in the water media. In the end, the NaBO_2_ was removed by washing it with DI water. The surfaces of the membranes that were electrospun using Ni^2+^ Cu^2+^/PVDF-HFP were green in hue. The chemical reduction in these metallic ions has a tendency to modify the color of their linked polymeric membranes into a black-look-alike black-dyed piece of fabric. This change in color is what indicated the formation of ultrasmall NiCu NPs on the surface of PVDF-HFP membranes.

### 2.4. Characterization

The fabricated catalytic NFs morphology was characterized by a scanning electron microscope (SEM, Hitachi S-7400, Japan) equipped with an energy-dispersive X-ray (EDX). A Fourier transform infrared (FTIR), using the smart ATR-FTIR model “Nicolet iS 10” (Thermo Fisher Scientific, Waltham, MA, USA) equipped with the specular reflectance, was also used to study the interaction between Ni Cu NPs and PVDF-HFP NFs. The fabricated NFs were positioned at the top of the spectrophotometer and the scanning range was 400–3500 cm^−1^. X-ray diffraction (Rigaku Co., Tokyo, Japan) with Cu Kα (λ = 1.54056 Å) was utilized to determine the catalysts’ crystalline structure and crystal size. An X-ray photoelectron spectroscopy analysis (XPS, AXIS-NOVA, Kratos Analytical, Manchester, UK) was conducted with the following conditions: base pressure of 6.5 × 10^−9^ Torr, resolution (pass energy) of 20 eV, and scan step of 0.05 eV/step.

### 2.5. SHB Catalytic Hydrolysis Reaction

To run the reaction experiments, a two-neck flask was used as a reactor. The reactor was placed on a magnetic stirrer. The magnetic stirrer provided both mixing and heating. The reaction temperature was measured by a thermometer. The flask was connected by a plastic tube to a gradually cylinder filled with water to measure the generated H_2_ gas. Water displacement from the gradually cylinder was reflected in the volume of H_2_ generation gas. To start the reaction, first, 100 mg of the prepared catalytic membranes was placed inside the flask. Then, 50 mL of 1 mmol of SBH was fed to the reactor and mixed at 100 rpm and the temperature was kept at 25 °C.

The catalytic hydrolysis reaction was started rapidly without induction time when catalytic NFs were conducted in the reactor. The kinetics of the hydrolysis reaction was studied by changing the amount of the catalysts, concentration of SBH, and temperature. To study the effect of a catalyst on the H_2_ production, different amounts of catalysts (100, 150, 200, and 250 mg) have been used. The effect of SBH concentration (1, 2, 3, and 4 mmol) on H_2_ production was also studied. To calculate the reaction activation energy, different reaction temperatures (298, 308, 318, and 328 K) have been studied. The durability of the introduced NFs was also studied through a recycling process. The reusability test has been conducted at 100 mg, 1 mmol, and 298 K of catalyst, SBH, and temperature, respectively. The catalyst has been used at all cycles without any washing. At each cycle, 1 mmol SBH was added and other parameters were kept constant.

## 3. Results and Discussion

**Figure 1:**
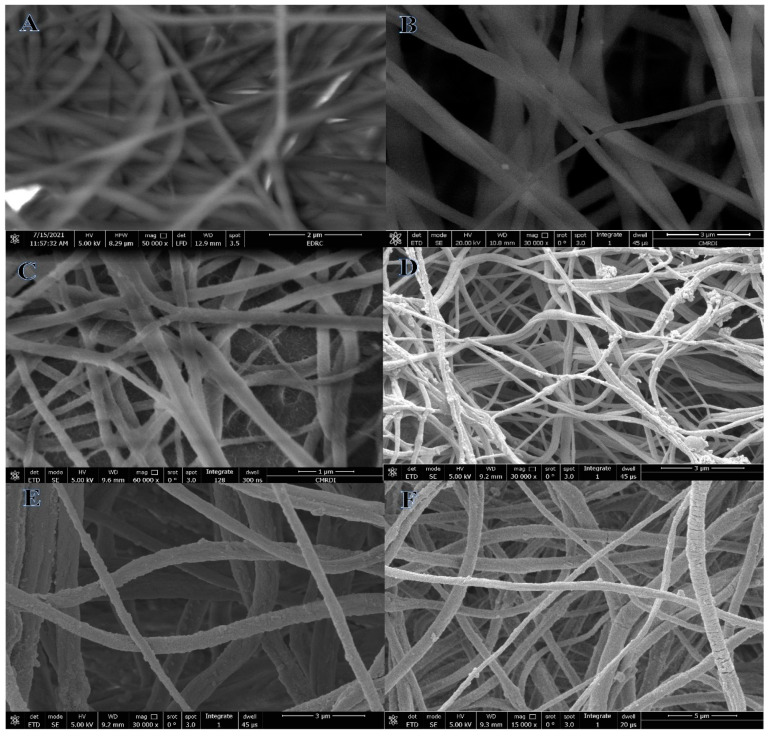
Low and high magnifications of SEM images PVDF-HFP (**A**), Ni_0.9_Cu_0.1_/PVDF-HFP (**B**), Ni_0.7_ Cu_0.3/_PVDF-HFP (**C**), Ni_0.5_Cu_0.5_/PVDF-HFP (**D**), Ni_0.3_Cu_0.7_/PVDF-HFP (**E**), Ni_0.1_Cu_0.9_/PVDF-HFP NF membranes (**F**).

Increased interconnections, flexibility, excellent porosity, and remarkable surface-to-volume ratios are just a few of the potential benefits of a polymeric nanofibrous membrane produced using the electrospinning approach [61,62]. The semi-crystalline nature, high thermal stability, improved dielectric constant, hydrophobicity, and piezo- and pyroelectric properties of PVDF-HFP make it one of the most suitable polymeric materials for producing these films [63,64]. SEM images of dried electrospun PVDF-HFP nanofibrous mats, at both low and high magnifications, proved the fabrication of an excellent nanofibrous structure devoid of beads (Figure 1A,B). It is well-known that the electrospinning technique produced nanoporous-structured NFs. These nanopores are generated in the electrospun NFs because of the high evaporation rate of solvents, especially acetone. Solvent evaporation happens when the NFs travel from the positive-charge electrode, syringe needle, to the negative electrode, rotating drum. This nanoporous structure is ideal for the nucleation of Ni and Cu crystals. In addition, since the CuAc and NiAc precursor salts have high water content, the produced hydrophobic PVDF-HFP membranes exhibit an increased demixing rate within the liquid–liquid phases. This demixing results in the formation of a high number of pores on their structure [65]. As a result, the analyte molecules would be able to get trapped in the pores with the minimal possibility of diffusion resistance, so making H_2_ evolution more straightforward. High electrical conductivity and gelation of the PVDF-HFP solution, together with the formation of maximal elongation of a jet along its axis, are two additional benefits of the presence of metal salts in the process of producing nanoscale polymeric NFs [44].

Figure 1C–F show SEM images of electrospun Ni_0.3_Cu_0.7_-PVDF-HFP and Ni_0.1_Cu_0.9_-PVDF-HFP NFs, respectively. Rough and bead-free NFs are created, as seen in the figure. In addition, the nanoporous nature of PVDF-HFP NFs means that reduced Ni and Cu ions may cover their surface after the mats are formed.

**Figure 2:**
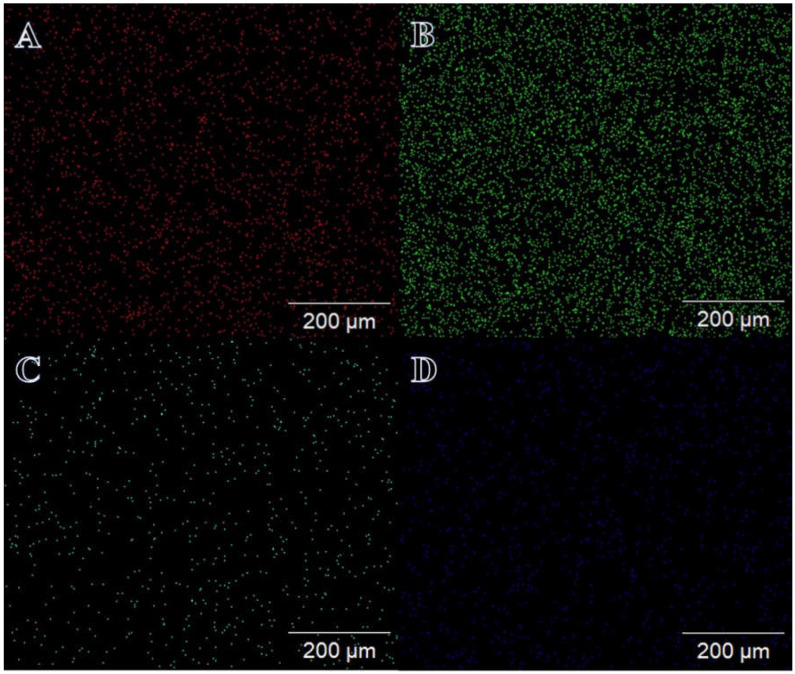
Elemental mapping to show the distribution of fluorine (**A**), carbon (**B**), nickel (**C**), and copper (**D**) in the Ni_0.3_Cu_0.7_/PVDF-HFP NF membrane.

**Figure 3:**
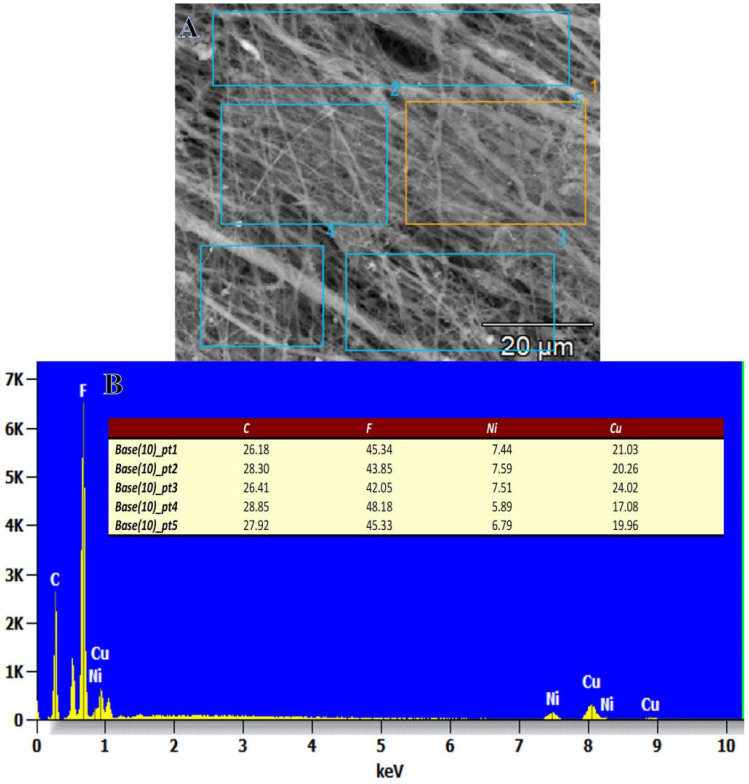
(**A**) SEM image and (**B**) EDX chart of Ni_0.3_Cu_0.7_/PVDF-HFP NF membrane at different SEM image points.

On display in Figure 2A–D is an elemental mapping image of the Ni_0.7_Cu_0.3_/PVDF-HFP membrane NFs. It is clear that there is a large distribution of Ni and Cu NPs throughout the membrane NFs, and this is supported by the pictures obtained from the SEM. The figure displays the EDX curve for the Ni_0.3_Cu_0.7_-PVDF-HFP membrane NFs (Figure 3A,B). The detected chemical composition of the product are the elements carbon, copper, nickel, and fluorine. The weight % of Ni and Cu that are developed at different points is shown in the inset of Figure 3B. It is obvious that NPs are distributed greatly throughout the NFs, and their composition is perfectly similar to that of the precursors from which they were originated.

**Figure 4:**
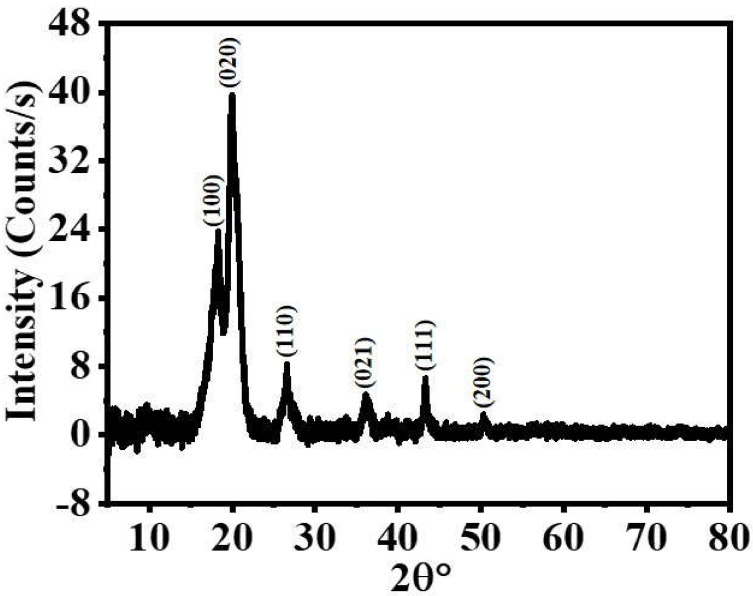
XRD results of Ni_0.3_Cu_0.7_/PVDF-HFP NF membranes.

Figure 4 shows the XRD diffraction patterns of the fabricated electrospun membranes. The Ni_0.3_Cu_0.7_/PVDF-HFP NFs, in its as-spun state, displayed characteristic diffraction peaks at 2 θ of 18.2°, 20°, 26.6°, and 36.15°, which correspond to the characteristic reflection planes of (100), (020), (110), and (021) [52,66]. The NiCu/PVDF-HFP catalyst had additional diffraction peaks at 43.4° and 50.4°, which are attributed to the (111) and (200) reflection planes, respectively. These planes are congruent with those of Ni (JCPDS 04-0850) and Cu (JCPDS, File no. 04-0836) as a result of their crystal structures being identical (face-centered cubic), of the very close crystal lattice parameters between Cu (3.615 Å) and Ni (3.523 Å), and of the same space group of Fm3m, which can be formed as a solid solution.

Using SBH as an effective reducing agent in a CH_3_OH medium, metallic Ni and Cu NPs were produced on the PVDF-HFP NFs surface. Because of their disparate reduction potentials (E_0_ Cu^++^/Cu^0^ = +0.34 V vs. SHE and E_0_ Ni^++^/Ni^0^ = −0.25 V versus SHE), SBH is more suited to reducing Cu ions to Cu^0^ than Ni ions to Ni^0^. First, the generation of Cu^0^ NPs stimulated SBH hydrolysis. Then, the production of an active intermediate, Cu-H species, becomes the key to facilitate Ni^++^ reduction. As a result, Cu^0^ plays a substantial role in improving the formation of the desired catalytic structure.

**Figure 5:**
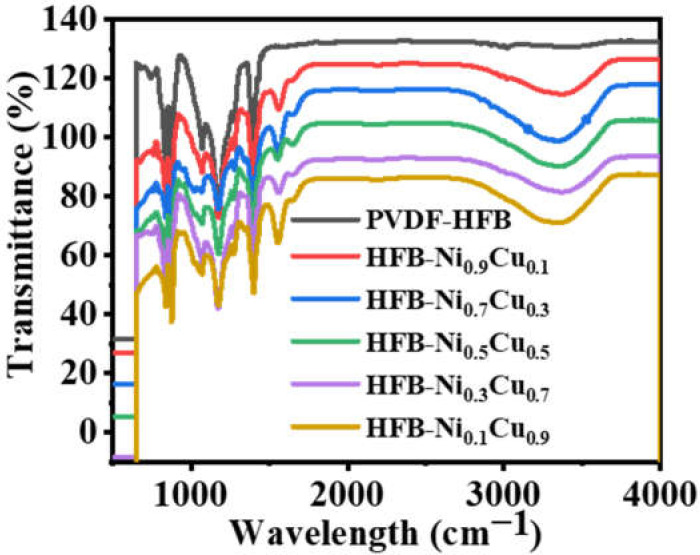
FTIR charts of PVDF-HFP and Ni_x_Cu_1−x_/PVDF-HFP NF membranes.

The FTIR spectra of PVDF-HFP and NiCu/PVDF-HFP fibrous mats are shown and explained in Figure 5. It is obvious that both fibrous mats had recognizable vibrational bands that were similar to one another. The phases of α and β were identified thanks to the corresponding peaks that occurred at frequency values of 749 and 837 cm^−1^ [67]. Two other important vibrational bands, centered at 672 and 872 cm^−1^, were designated for the CF and CH_2_ wagging of vinylidene units in the amorphous phase of the PVDF-HFP film. These bands were found in the amorphous phase of the film. Additionally, the symmetric C-F stretching, the CF_2_ stretching, and the deformed vibrations in this fibrous mat were also revealed via their respective bands at 1071, 1175, and 1400 cm^−1^ [68]. During the process of fabricating the polymeric membrane, the addition of nickel and copper precursor salts caused the appearance of two more peaks. The stretching vibration maxima at 1561 cm^−1^ proved the production of NiO and CuO species inside this nanomaterial [69].

### Hydrogen Release from SBH

**Figure 6:**
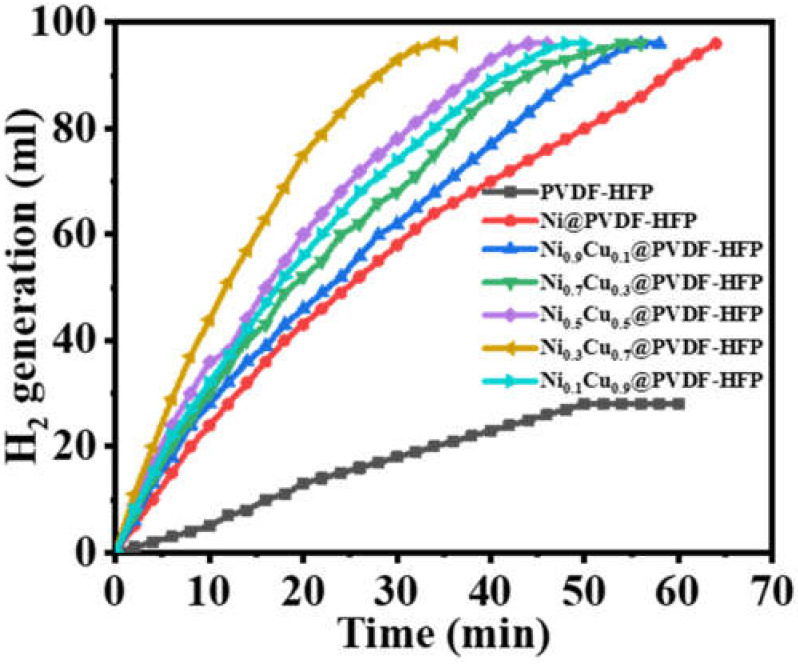
Influence of Ni_x_Cu_1−x_/PVDF-HFP NF membranes on H_2_ production from SBH hydrolysis at 100 mg catalyst, 1 mmol [SBH], and 298 K.

In a two-neck flask connected to a graduated burette containing water, the catalytic activity of the fabricated catalysts (Ni/PVDF-HFP and Ni_x_Cu_1−x_/PVDF-HFP) toward H_2_ production from SBH was determined. Figure 6 shows the catalytic activity of various Ni_x_Cu_1−x_/PVDF-HFP formulations compared to that of the mono-metallic Ni/PVDF-HFP; the bimetallic alloys display a high hydrogen-production volume. The H_2_ production rate initially grew and subsequently fell over the course of a given time period. The catalytic reaction in this case seems to take place on the metal catalyst’s surface [19]. These findings provide strong evidence for a significant role for the synergistic interaction between Cu and Ni in the hydrolysis of SBH. The synergistic effects of NPs alloy can be attributed mostly to the electronic effects, which become significant when the metal atoms have considerable variances in electronegativity and geometric effects [20]. The electronegativities of Cu and Ni are so similar: 1.90 and 1.91, respectively; therefore, it is likely that the obtained synergetic effects of the fabricated Ni_x_Cu_1−x_/PVDF-HFP catalysts are mostly attributable to the geometric effects rather than the electronic effects. Since both Cu and Ni have an A1-type crystal structure, the formation of CuNi alloy NPs allows Ni to replace part of the Cu in the Cu lattice while still allowing the Cu crystal system to maintain its A1-type crystal structure, resulting in strong synergistic effects between Cu and Ni. The bimetallic catalyst Ni_0.3_Cu_0.7_ showed the maximum catalytic activity, releasing 96 mL of H_2_ in 36 min. The other four bimetal catalysts (Ni_0.9_Cu_0.1_, Ni_0.7_Cu_0.3_, Ni_0.5_Cu_0.5_, and Ni_0.1_Cu_0.9_) catalyzed H_2_ release equivalents of 71, 79, 87, and 83 mL, respectively, in the same time period. In terms of catalytic activity, the order of the engaged bimetal catalysts is as follows: Ni_0.3_Cu_0.7_ > Ni_0.5_Cu_0.5_ > Ni_0.1_Cu_0.9_ > Ni_0.7_Cu_0.3_ > Ni_0.9_Cu_0.1_. According to the findings, the NiCu’s catalytic activity changes when the Cu/Ni ratio is altered. When comparing the catalytic activity of all five bimetal catalysts, it is found that as the percentage of Cu increases, the catalysts become more effective. It is theorized that an increase in the Cu content of NiCu alloy facilitates the passage of more electric charge from Cu to Ni, resulting in the generation of more active sites on the catalytic surface. The greater catalytic activity of bimetallic alloy in comparison to mono metals have been observed in other metal catalysts [21,22,23,24,25,26]. For example, Li [27] and Yamada [28] have independently discovered that the Cu concentration of a Cu-based bimetal catalyst considerably influences its catalytic activity. Furthermore, the low catalytic activity and nearly nonexistent catalytic activity of pure PVDF-HFP toward hydrolysis of SBH are readily apparent. These findings give conclusive evidence that the bi-functional impacts of CuNi-alloy NPs and PVDF-HFP considerably improved the reaction activity of the Ni_0.3_Cu_0.7_/PVDF-HFP catalyst, where the catalytic support PVDF-HFP NFs may serve as a suitable matrix for CuNi NPs despite their lack of involvement in SBH hydrolysis. The mechanism involved two main steps: at first, SBH is adsorbed on the CuNi nanoparticles’ surface to produce the activated intermediate metal-H, a condition precedent to the hydrolysis process. Then, when water was thrown at the metal-H species, H_2_ was generated [29,30].

**Figure 7:**
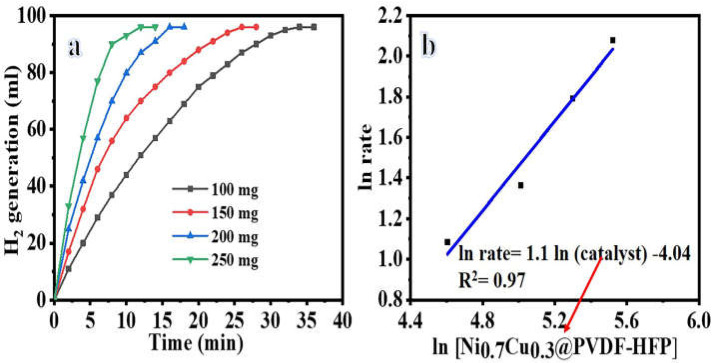
The effect of changing the amount of Ni_0.3_Cu_0.7_/PVDF-HFP NFs on H_2_ production (**a**), and the H_2_ production rate logarithmic value vs. at catalyst amount logarithmic value (**b**) at 1 mmol [SBH] and 298 K.

The performance of the fabricated nanofibrous catalysis is a function of several factors including SBH concentration, reaction temperature, catalyst amount, and catalyst reusability. Therefore, the effect of operating conditions on the performance of the best fabricated catalyst, Ni_0.3_Cu_0.7/_PVDF-HFP, was studied. First, the effect of the amount of the catalyst on H_2_ generation, at 25 °C and 1 mmol of SBH, was studied. Increases in the catalyst amount, as shown in Figure 7a, resulted in an increase in the generated H_2_ volume. In fact, increasing the catalyst dose from 100 mg to 250 mg shortened the time it took for H_2_ evolution from 36 minutes to 12 minutes. So, for all reactions carried out under similar conditions, the time required to produce H_2_ reduces practically linearly as the amount of catalyst used increases. While this relationship is only somewhat linear, it does show that the hydrolysis process is first-order with regard to the amount of catalyst within a slope of 1.1 (Figure 7b). As a result, 100 mg of Ni_0.3_Cu_0.7_/PVDF-HFP was selected for use in the following studies.

**Figure 8:**
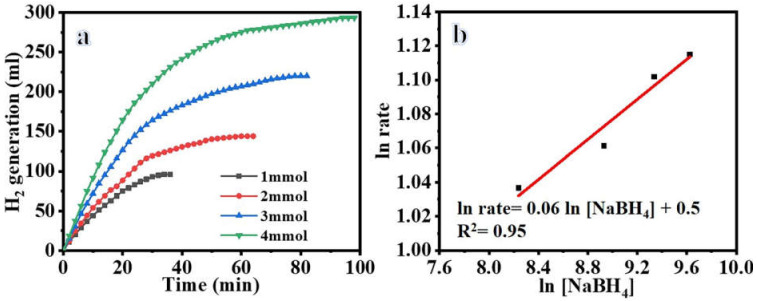
The effect of SBH concentration on H_2_ production (**a**), and the H_2_ production rate logarithmic value vs. [SBH] concentration logarithmic value (**b**) at 100 mg catalyst and 298 K.

Figure 8a shows the results of an experiment that investigated the impact of SBH concentration on reaction rate using 100 mg of Ni_0.3_Cu_0.7_/PVDF-HFP catalyst and a temperature of 25 °C. The rate of produced H_2_ at the outset increases linearly with the concentration of SBH, as shown in the figure. This demonstrates that the volume of H_2_ generation does not rely on the amount of SBH present. Since the start, SBH concentration has no effect on the hydrolysis process, and the slope of 0.06 indicates that the hydrolysis reaction is in zero order.

**Figure 9:**
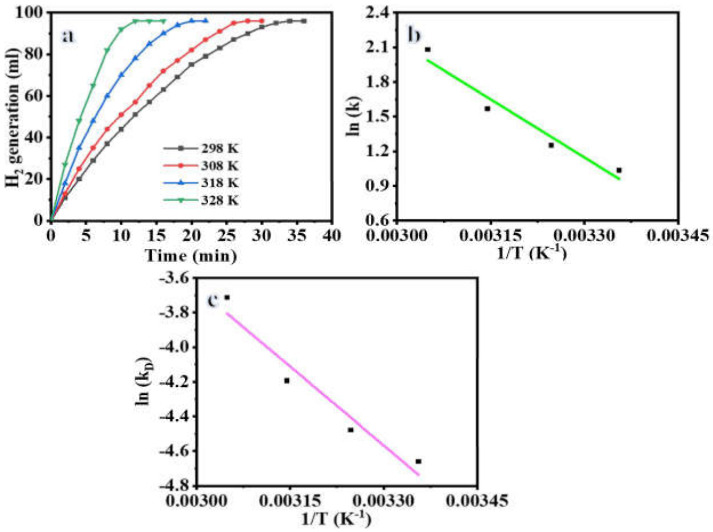
Influence of reaction temperature on H_2_ production (**a**), the H_2_ production rate constant logarithmic value vs. (1/T) (**b**), and K_D_ (K/T) logarithmic value vs. (1/T) (**c**) at 100 mg catalyst and 1 mmol [SBH].

The catalytic performance of the Ni_0.3_Cu_0.7_/PVDF-HFP catalyst may be affected by the reaction temperature, which in turn affects the chemical kinetic characteristic of the H_2_ release process. To further understand this, 100 mg of Ni_0.3_Cu_0.7_/PVDF-HFP catalyst and 1 mmol of SBH were used to conduct experiments at four different reaction temperatures (298 K, 303 K, 308 K, and 328 K). Figure 9a shows that when the reaction temperature is raised, the reaction time for H_2_ generation decreases. The values of the rate constant, k, can be determined by examining the slope of the linear portion of the plots at various reaction temperatures. Figure 9b displays the ln k vs. 1/T plot that was constructed using the activation parameters for the hydrolysis processes of SBH utilizing Ni_0.3_Cu_0.7_/PVDF-HFP catalyst, which were determined using the well-known Arrhenius and Eyring equations. For the Ni_0.3_Cu_0.7_/PVDF-HFP catalyst system, the activation energy for the hydrolysis process of SBH solution was determined to be Ea = 27.81 kJ/mol. The activation energy of the non-noble metal catalyst Ni_0.3_Cu_0.7_/PVDF-HFP is low when compared to that of Ni-based catalysts and Cu-based catalysts as shown in Table 1. It seems that the fabricated Ni_0.3_Cu_0.7_/PVDF-HFP catalyst achieves excellent catalytic performance in SBH hydrolysis.

**Figure 10:**
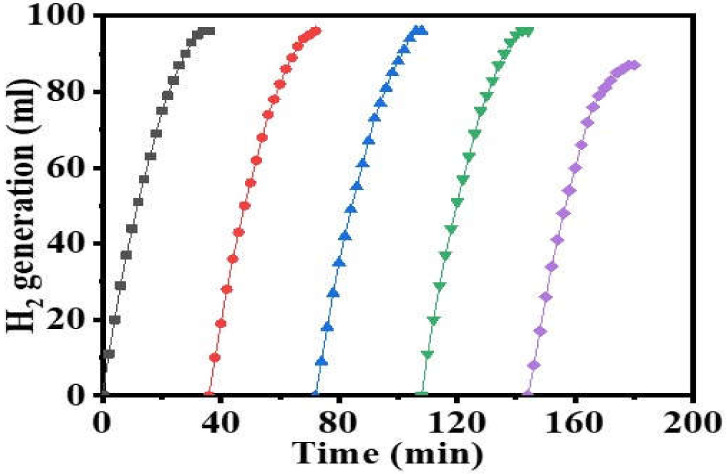
Reusability test of Ni_0.7_Cu_0.3_@PVDF-HFP NF membranes. (The amount of catalyst = 100 mg, (SBH) = 1 mmol, and T = 298 K.)

To evaluate the catalytic reusability, the same catalyst (100 mg of Ni_0.3_Cu_0.7_/PVDF-HFP) was utilized up to five times in the reduction processes of 1 mmol SBH at 25 °C with a mixing rate of 1000 rpm (Figure 10). The percentage of conversion remained the same as indicated in Figure 10, but the activity of the catalysts decreased to 93% after four cycles. The remarkable durability should be ascribed to the crystalline structure of the catalyst, which has remained mostly constant, as well as the ultrafine CuNi NPs, which have continued to be well-stabilized at PVDF-HFB throughout the catalytic cycles. It was important to point out that the SBH was introduced to the reactor without separating the catalytic NFs. At the end of each cycle, the hydrolysis process of SBH will have been finished completely. The decrease in catalytic activity, which began after four runs, may be attributed to the following: (1) an increase in the solution viscosity and (2) the passivation of the NFs’ surface, which may be attributed to the accumulation of boron products (such as metaborate) on the reaction solution and polymer surface, which inhibited the metal active sites [12].

Equations (3)–(5) were applied to express SBH hydrolysis kinetics as a function of Ni_0.3_Cu_0.7_/PVDF-HFP concentration, SBH concentration, and reaction temperatures.

(3) $$r=-d\left[SBH\right]dt=k{\left[HFB-40\right]}^{1.1}\left[SBH^{-0.06}\right]$$

(4) $$k=Ae^{\left(\frac{-E_{a}}{RT}\right)}\to \;\ln k=\ln12.188-\frac{3345.9}{8.314T}$$

(5) $$r=-d\left[SBH\right]dt=12.1882e^{\left(-3345.9/T\right)}{\left[HFB-40\right]}^{1.1}\left[SBH^{-0.06}\right]$$

The activation enthalpy (ΔH, (KJ/mol)) and the activation entropy (ΔS, (J/mol.K)) may be used to get the Gibbs free energy of activation (ΔG, (KJ/mol)) using Equations (6) and (7).

(6) $$\ln k_{D}=\ln\frac{k_{B}}{h}+\frac{\Delta S}{R}-\frac{\Delta H}{RT}$$

(7) ΔG=ΔH−TΔS

where k_D_ = (k/T), K_B_ is Boltzmann constant (1.381 × 10^−23^ J·K^−1^), and h is the Planck constant (6.626 × 10^−34^ J·s^−1^). According to Equation (7) in Figure 9c, ΔH and ΔS is estimated to be 33.83 kJ mol^−1^ and 0.074 kJ mol^−1^, respectively. Hence, the ΔG equation can be summarized as follows: ΔG=33.83−0.074T.

## 4. Conclusions

CuNi nanoparticles supported on PVDF-HFP nanofibers catalysts have been effectively fabricated by the electrospinning technique and in situ reduction in metal ions. The fabricated bimetal catalysts have presented a superior catalytic performance in H_2_ generation from SBH as compared to monometallic counterparts. The alloy composed of Ni_0.3_Cu_0.7_/PVDF-HFP NFs has shown good catalytic activity as compared to other formulations. The excellent catalytic activity is in regard to the synergistic effects between Ni and Cu resulting from their geometric effects and uniform distribution of bimetallic NPs. According to the findings of the kinetics investigations, the reaction follows a zero-order and a first-order mechanism with regard to the SBH concentration and the catalyst amount, respectively. In addition, a low activation energy, evaluated as Ea = 27.81 kJ/mol, was obtained for the hydrolysis process that was performed on the SBH solution.

The catalyst has been reused for five cycles. The great catalytic performance and simple separation of the introduced catalytic NFs make them a promising candidate to be a highly efficient catalyst for a hydrogen storage system that enhances commercial applications.

## Figures and Tables

**Table 1:** Activation energies of Ni- and Cu-based catalysts for H_2_ generation from SBH.

Catalyst	Ea (KJ/mol)	Ref.
Ni	42.28	[70]
Ni	71	[71]
Raney Ni	63	[71]
Ni-Ag	16.2	[72]
Ni-Co	38	[73]
Ni-Co-B	62	[74]
Co-B/Cu	43.3	[75]
Co-Cu-Ni	40.6	[76]
Co-Cu-B/Ni foam	52	[77]
WSC-Cu	32.7	[78]
P(EP-g-AA)-Cu	42.6	[79]
Co-Ni/AC	68.9	[19]
Co-Ni/MWAC	40.7	[80]
Sm- Ni-Co-P/g-Al2O3	52.1	[81]
Ni–Co–B	62	[75]
Ni-hollow PVDF capsules	49.3	[65]
Ni-PVDF hollow fiber	55.3	[82]
([C6(mpy)2][NiCl4]2-	56.4	[83]
PVDF-[C6(mpy)2][NiCl4]2-	44.6	[41]
NiCu/PVDF-HFP	27.8	This study

## Data Availability

Not applicable.
